# Report of Multilocus Inherited Neoplasia Alleles Syndrome in a Chilean Oncology Institute: New Combinations and Genetic Landscape

**DOI:** 10.3390/genes17070839

**Published:** 2026-07-22

**Authors:** Francisca Sepúlveda Bustos, Fernanda Martin Merlez, Danitza Campos Jadrijevic, María Paz Saavedra, Carolina Selman Bravo

**Affiliations:** 1 Sección Genética, Departamento de Medicina, Hospital Clínico Universidad de Chile, Santiago 8380456, Chile; 2 Unidad Asesoramiento Genético Oncológico, Fundación Arturo López Pérez OECI Cancer Center, Santiago 7500000, Chile; 3 Unidad de Genética, Hospital del Salvador, Santiago 7500000, Chile; 4 Subgerencia Unidades de Diagnóstico, Fundación Arturo López Pérez OECI Cancer Center, Santiago 7500921, Chile

**Keywords:** MINAS, hereditary cancer, genetic counseling, Chile

## Abstract

**Background/Objectives**: Multilocus Inherited Neoplasia Alleles Syndrome (MINAS) is defined by the presence of germline pathogenic or likely pathogenic variants in two or more distinct cancer susceptibility genes (CSGs) in the same individual. Although carriers may present more complex phenotypes, the clinical and molecular spectrum of MINAS remains poorly characterized, particularly in underrepresented populations. We aim to describe this phenomenon in a cohort of individuals from a Chilean institution. **Methods**: We retrospectively reviewed individuals evaluated at the Oncogenetic Counseling Unit of Fundación Arturo López Pérez (FALP) between 2020 and 2026 who underwent hereditary cancer multi-gene panel testing. Cases fulfilling MINAS criteria were described. We analyzed the association between MINAS and age at cancer diagnosis or multiple primary cancers, and reviewed reported cases with the same gene combinations. **Results**: From 1962 individuals tested, 398 harbored a pathogenic or likely pathogenic variant, and 14 fulfilled MINAS criteria, yielding a prevalence of 3.51% among positive cases. Breast cancer was the most common tumor type (76.9%), and *ATM* and *CDKN2A* were the most frequently involved genes. MINAS was significantly associated with a younger age at cancer diagnosis, but not with multiple primary cancers. **Conclusions**: MINAS prevalence in our cohort and the association with a younger diagnosis of cancer was consistent with published series. We identified seven previously unreported gene combinations, and common founder variants shifted the pattern away from predominantly BRCA-associated combinations. Despite the small sample size, this study adds relevant data from an underrepresented Latin American population.

## 1. Introduction

Multilocus Inherited Neoplasia Alleles Syndrome is defined as the presence of germline pathogenic or likely pathogenic variants in two or more distinct cancer susceptibility genes in the same individual, excluding biallelic variants in the same gene [1]. The term was coined by Whitworth and colleagues in 2016, who described a series of five original cases alongside a systematic review of 82 additional cases from the literature, identifying 17 implicated genes and proposing for the first time the need for a centralized international registry in the LOVD [2,3].

The identification of individuals with MINAS has increased considerably over the past decade, in direct relation to the expanded use of next-generation sequencing (NGS) multi-gene panels, whole-exome sequencing (WES), and whole-genome sequencing (WGS). This primarily reflects increased detection rather than a true change in the incidence of the condition [4]. By 2022, at least 385 individuals with MINAS had been reported worldwide, involving pathogenic variants in 63 different CSGs, a number that rose to 413 cases in the most recent review [5].

The prevalence of MINAS varies according to population and methodology, ranging from 1.37% reported in Spanish cohorts to 5.9% described in the Mexican population, with intermediate figures in series from the United States (~3%) and China (~2.16%) [4,6]. The most frequently involved genes are *BRCA1* and *BRCA2*, present in 78.5% of reported cases, followed by *CHEK2*, *ATM*, and the mismatch repair genes (*MLH1*, *MSH2*, *MSH6*, *PMS2*) [4].

The effects of MINAS remain poorly characterized; however, recent publications consistently suggest that MINAS carriers may present a more complex and severe phenotype, including multiple primary neoplasms, an earlier age of onset, or tumors not strictly related to the phenotype associated with either individual pathogenic variant [5,7].

Despite the considerable growth in knowledge about MINAS in recent years, as well as the increasing number of publications related to this condition, most available data originate from North America and Europe. This creates an important gap in information from underrepresented populations, limiting the understanding of the phenotypic and genotypic spectrum of MINAS, as well as its variability across different populations [7]. It is therefore essential to characterize underrepresented groups, such as the Chilean population, in order to contribute to a broader understanding of this condition.

In this study, we describe the clinical and molecular characteristics of patients with MINAS evaluated in an oncogenetic counseling unit in a Chilean oncology institute named Fundación Arturo López Pérez (FALP) between 2020 and 2026.

Individuals were referred to this genetic counseling unit via direct consultation with their treating physicians within the same institution, referral by the oncology committee, or—in some cases—self-referral. All patients went through a three-step process: 1. an interview with a nurse to review the unit’s oncology questionnaire results and create a three-generation family tree; 2. a pre-test interview with a geneticist or a breast surgeon trained in genetic counseling; and 3. a post-test interview with a geneticist or a breast surgeon trained in genetic counseling to provide results and recommendations. Genetic testing was indicated based on National Comprehensive Cancer Network criteria.

## 2. Materials and Methods

We conducted a cross-sectional retrospective study of individuals evaluated at the genetic counseling unit of FALP between 2020 and 2026 who underwent genetic multi-gene panel testing for hereditary cancer, searching for positive cases with pathogenic/likely pathogenic variants (PV/LPVs) in more than one CSG.

Multi-gene panel testing was carried out in Clinical Laboratory Improvement Amendments (CLIA)/College of American Pathologists (CAP)-certified laboratories in the USA, Brazil or Chile, with Illumina short-read sequencing technology from blood or saliva samples. All individuals were provided with pre- and post-test counseling.

Fisher’s exact test was used to examine relationships between categorical variables such as the presence of MINAS or the nature of identified MINAS genes (dominant–dominant, dominant–recessive) with phenotypic characteristics: age at first cancer diagnosis (>30 or >40), more than one primary cancer diagnosis or atypical phenotypes; the presence of MINAS with a positive family history of cancer (>2 individuals in the family with related cancers as stated in NCCN guideline criteria) was the indication for testing.

We conducted a review of previous reported cases of MINAS with the same gene combinations as the individuals in this study in the MINAS Leiden Open Variation Database (LOVD) [2,3], the review of MINAS by Yuen et al. 2025 [5] and a search on PubMed for the terms “double heterozygosity” OR “double heterozygote” OR “double mutation” OR “double heterozygous mutation” OR “double germline mutation” OR “digenic” OR “MINAS” OR “Multilocus Inherited Neoplasia Alleles Syndrome” in the title or abstract between the years 2023 and July 2026. The resulting articles within the search were then filtered manually (FM) by their title to assess if they included cases of MINAS.

## 3. Results

Between 2020 and 2026, a total of 1962 individuals attending the genetic counseling unit at FALP underwent hereditary cancer multi-gene panel testing (Supplementary Tables S1 and S2). Of the tested individuals, 55% were patients from FALP, while the remainder were referred from other public and private institutions within the country. Although the vast majority of the patients treated were Chilean, no analysis was conducted to verify ancestry. A total of 63% of the tested patients were covered by the national public health insurance (FONASA) and the remainder had private insurance, whereas 85% of the Chilean population belongs to FONASA.

Overall, 95% were female and 83% had a diagnosis of cancer, with breast cancer (BC) accounting for 67% and ovarian cancer for 3% of these diagnoses (Supplementary Tables S3–S5). Of these, 398 (20%) had a positive result with a PV/LPV identified, and 14 individuals were found to carry variants in more than one cancer predisposition gene, consistent with MINAS, representing a prevalence of 3.51% among PV/LPV carriers. The clinical and molecular characteristics of all cases are summarized in Table 1.

### 3.1. Clinical and Demographic Features of MINAS Cases

Ages at the time of assessment ranged from 17 to 52 years, with a median age at first cancer diagnosis of 35.6 years. All individuals were female. The predominant phenotype was breast cancer, present in 10 of 13 cases with cancer (76.9%), frequently with early onset (≤45 years). Additional phenotypes included paraganglioma (*n* = 1), multiple melanomas (*n* = 2), mismatch repair-deficient colon adenocarcinoma (*n* = 1), and a family history of pancreatic cancer (*n* = 1). Two individuals with MINAS (14.2%) had more than one different primary malignancy throughout their clinical history (cases 6 and 8).

We considered case 12 to have an atypical phenotype. Even though there are some reports that describe a possible association between the *CDKN2A* PV/LPV with breast cancer risk [8,9], it is not considered an actionable gene for this type of cancer [10,11].

We deemed cases 3, 6, 8, and 9 as cases with a more severe phenotype than expected for the spectrum of those genes. Cases 3 and 9 involved a primary diagnosis of breast cancer before age 30; case 6 involved stage IV colorectal cancer at 38 yrs old in a patient from a family with only one previous case of colorectal cancer at a more advanced age; and case 8 involved a diagnosis of breast cancer at 37 and ovarian cancer at 41 in a moderate–low penetrance gene for breast and ovarian cancer respectively.

There was a significant statistical association in this cohort between the diagnosis of MINAS and the diagnosis of cancer at age 30 or younger (OR = 7.9, *p* = 0.01, 95% CI: [1.8338, 29.5401]), or at age 40 or younger (OR = 3.8, *p* = 0.03, 95% CI: [1.1243, 12.8637]), and we found no association between the presence of more than one primary cancer diagnosis and MINAS (OR = 1.2, *p* = 1, 95% CI: [0.2340, 5.0250]), and a positive family history as the motive for testing (OR = 0.9, *p*= 1, 95% CI: [0.3289, 2.8343]).

### 3.2. Molecular Findings

The following gene combinations were identified (Table 2): *BRCA1/CDKN2A* (*n* = 1), *BRCA1/PMS2* (*n* = 1), *SDHB/RECQL4* (*n* = 1), *APC/MUTYH* (*n* = 1), *FLCN/CDKN2A* (*n* = 1), *ATM/MUTYH* (*n* = 1), *CDKN2A/LZTR1* (*n* = 1), *ATM/FANCM* (*n* = 1), *BRCA2/FANCA* (*n* = 1), *BRCA2/ATM* (*n* = 1), *CHEK2/ATM* (*n* = 1), *BRCA2/MUTYH* (*n* = 1), *ATM/MLH1* (*n* = 1), and *CDKN2A/MUTYH* (*n* = 1). There were no cases with more than two PV/LPVs identified.

Combinations involving *ATM* were the most frequent, accounting for 35% of cases, followed by individuals carrying the *CDKN2A*:c.-34G>T splice variant which was present in four cases (28.5%) or variants of *MUTYH* (28.5%). MINAS cases involving *BRCA1* or *BRCA2* variants represented 35%, with no individuals carrying variants of these two genes simultaneously.

Regarding the nature of the variants, two categories were distinguished: combinations in which both variants confer autosomal-dominant predisposition to cancer (D-D), observed in seven cases (50%); and combinations involving one dominant and one recessive variant (D-R) (50%). There were no cases with two recessive variants (R-R) identified in this cohort.

There was no statistically significant association between the combination of the nature of variants (D-D or D-R) and the presence of an atypical phenotype considering case 12 as atypical (OR = 4.09, *p* = 0.46, 95% CI: [0.1387, 120.6971]); nor with the diagnosis of more than one primary cancer (OR = 0.8, *p* = 1, 95% CI: [0.0409, 16.9953]).

### 3.3. Previous Reports of the Same MINAS Combinations

A systematic search of the MINAS LOVD [3] and PubMed was conducted for each of the 14 gene combinations identified in this cohort (Table 3). Of these, seven combinations were previously reported in the literature or registered in the LOVD. The remaining seven combinations (*BRCA1/PMS2*, *ATM/MLH1*, *ATM/FANCM*, *CDKN2A/FLCN*, *CDKN2A/LZTR1*, *CDKN2A/MUTYH*, and *SDHB/RECQL4*) had no prior reports in the literature or entries in the LOVD, representing novel MINAS combinations.

## 4. Discussion

In this series, we describe 14 MINAS cases identified at an oncogenetic counseling unit in Chile between 2020 and 2026, representing a prevalence of 3.51% among PV/LPV carriers. This number is comparable to that reported in series from the United States (~3%) and China (~2.16%), higher than described in Spanish cohorts (1.37%), and lower than the Mexican series (5.9%) [4,6]. Multiple heterozygosity was likewise reported in 2.9% of carriers of HBOC-associated PV in the German Consortium for Hereditary Breast and Ovarian Cancer (GC-HBOC) registry, a figure the authors regard as a lower-bound estimate given the restriction to 13 core genes [20].

The variability across populations may reflect differences in the multi-gene panels used, the criteria for genetic testing referral, and the genetic composition of each population.

The predominant phenotype in our cohort was breast cancer, present in 76.9% of cases, which is consistent with international series, where *BRCA1* and *BRCA2*—genes primarily associated with breast and ovarian cancer—account for 78.5% of reported MINAS cases [4]. However, unlike global and other Latin American series, *ATM* and *CDKN2A* were the most frequently involved genes in our cohort, while *BRCA1* and *BRCA2* were present in a smaller proportion of cases.

Previous reports of MINAS in Latin American populations were mainly derived from HBOC cohorts. In the Mexican series, 95.6% of MINAS cases were identified among HBOC patients [6]; and in a Brazilian study including 1663 breast cancer patients, although MINAS was not specifically evaluated, 18 individuals carried more than one PV/LPV, of whom 55% had a *BRCA1/2* variant [21]. Although breast cancer was also the predominant phenotype in the population tested in this study (67%), this proportion was lower than in those studies, reflecting the inclusion of patients referred for other hereditary cancer syndromes. This may explain the lower frequency of *BRCA1/2* combinations observed in our series, since the prevalence of *BRCA1/2* pathogenic variants among Chilean individuals undergoing HBOC testing is comparable to that reported in other South American and U.S. Hispanic populations [23,24].

Regarding *BRCA1* and *BRCA2*, it is worth noting that the Chilean population has a distinct mutational landscape compared to other Latin American countries. Nine founder variants were identified by haplotype analysis accounting for 78% of *BRCA1/2* variant carriers [25]. However, subsequent studies including a national literature review observed a lower frequency of 51.9% for these variants among Chilean families [23]. The variants identified in our cohort in cases 1, 2 and 3 (*BRCA1* c.3759dup, *BRCA1* c.3331_3334del, and *BRCA2* c.4740_4741dup) are among the founder mutations described for the Chilean population.

The frequent presence of the *CDKN2A c.-34G>T* variant might be explained by the amplitude of the multi-gene panel test used in each cohort since *CDKN2A* is not usually included in HBOC genetic panels, but it is in multicancer panels, which were the most frequently solicited in this cohort (78%). This variant, present in four of our MINAS cases (cases 1, 10, 11 and 12), is the most recurrent *CDKN2A* variant in Latin America according to a multicenter study including patients from Argentina, Brazil, Chile, Mexico and Uruguay. This variant was found at high frequency among unrelated Chilean families (90% of *CDKN2A*-mutated families), suggesting a possible founder effect in this population [26].

In the Brazilian report on MINAS cases within a breast cancer patient cohort, all patients were tested using a 20-gene panel that did not include *CDKN2A*; testing for this gene was conducted only at the treating physician’s discretion, without specifying the cases in which it was requested [21]. In the Mexican report on MINAS, individuals were evaluated using various panels comprising 7, 30, 35, and 84 genes, but the specific gene content analyzed in the cases was not specified; consequently, no conclusions can be drawn regarding the founder effect hypothesis or the presence or absence of the gene in the panels evaluated [6].

The absence of *TP53* in our MINAS cohort contrasts with the Brazilian series [21], where the *TP53 p.R337H* variant is exceptionally prevalent due to a well-documented founder effect. Population-based newborn screening studies have established that this variant is present in approximately 0.21–0.30% of the population in South and Southeast Brazil, corresponding to 1 in 300–500 individuals [27,28,29]. This variant exhibits incomplete penetrance and is associated with an attenuated Li–Fraumeni phenotype, with cancer risk further modulated by the co-inherited XAF1 p.E134* variant present in approximately 69% of carriers [30,31]. The distinct population genetics in Chile, where no *TP53* founder effect has been reported, likely explains the absence of this gene in our MINAS cohort.

Regarding *MUTYH*, the two pathogenic variants that have been reported as most frequent in Latin America are *p.Gly396Asp* (also known as G368D, as reported in this cohort) and *p.Tyr179Cys*, both of European founder origin, present in approximately 78% of cases in a multicenter Latin American series, similar to what has been reported in Caucasian populations [32]. In our cohort, we identified four MINAS cases with *MUTYH* variants (cases 5, 7, 12 and 14), three of which carried the *p.Gly396Asp* variant and one the *p.Tyr179Cys* variant. Notably, three of these four cases were breast cancer, which is concordant with the higher proportion of breast cancer compared to colorectal cancer (46.1% vs. 24.7%) observed in monoallelic *MUTYH* carriers in Latin America [32]. The carrier frequency for heterozygous *MUTYH* variants in European-descent populations is 1–2%; however, no specific data on population carrier frequency in Chile are available.

These findings suggest a relevant influence of the basal genetic composition of different populations in MINAS diagnosis, with founder variants appearing in this cohort in a significant proportion of cases, not necessarily related to the primary phenotype. Given the association found between a younger age of diagnosis and the phenomenon, it would be reasonable to always consider multi-gene panel tests that include genes with founder variants in a given population, regardless of the phenotype, to not miss potential cases of MINAS which we now know might have phenotypic consequences [5].

In MINAS with previous reports, the most described combination (*BRCA2/ATM*) with more than fifteen cases, as with the *BRCA2/MUTYH* combination with four previous cases, seems to follow a “typical” *BRCA2* pattern, with early onset of breast cancer accompanied in some cases by a second breast cancer diagnosis and pancreatic cancer [33]. In all the other cases the scarcity of phenotypic reports makes any assumption about the combination of those genes very difficult. In our cohort the *BRCA1/CDKN2A*, *BRCA1/PMS2*, *SDHB/RECQL4*, *ATM/MUTYH*, *CDKN2A/FLCN* and *CDKN2A/LZTR1* MINAS tend to resemble the phenotypic spectrum of the dominant gene with the higher penetrance, taking into account the one-point-in-time description and the uncertainty that they might develop other cancers in the future.

Recent evidence from the GC-HBOC registry has refined the understanding of these dominant-gene patterns by providing genotype–phenotype data for specific combinations [20]. Of particular interest to our cohort, *ATM/CHEK2* double heterozygotes presented a median age at first breast cancer diagnosis of 41.5 years (IQR 14.0), earlier than single *ATM* (46.1; IQR 13.2) or *CHEK2* carriers (47.6; IQR 14.9), and comparable to single *BRCA1* carriers (41.3; IQR 14.1).

Our case 9, involving this same combination, was diagnosed at age 29, representing a markedly earlier onset than isolated variants would predict. These data support our finding of an association between MINAS and younger age at diagnosis, suggesting that certain moderate-penetrance gene combinations might justify earlier breast surveillance. However, it is important to note that these phenotypic differences did not achieve statistical significance in the German study, and their cohort was limited to breast and ovarian cancer genes within a population whose founder variant landscape differs from the Chilean landscape.

Returning to the *BRCA2/ATM* combination discussed above, the GC-HBOC registry provides phenotypic data. *BRCA2/ATM* double heterozygotes had a median age at first breast cancer of 40.1 years (IQR 12.1), placing them among the earliest-onset combinations described [20]. Our case 4 developed breast cancer at 41 years, in agreement with that median. However, while *BRCA2*-containing combinations in the German series showed low triple-negativity rates and were predominantly HR-positive and HER2-negative, our case presented with a triple-negative tumor, illustrating the phenotypic variability still to be expected within individual gene combinations. Taken together, these data suggest that specific gene–gene constellations may reshape the tumor phenotype and disease severity beyond single-gene expectations, while the variability observed within each combination indicates that this effect is not uniform across carriers.

In a Chinese high-risk breast cancer cohort of 3649 patients, eight MINAS carriers were identified, seven of whom carried a *BRCA1* variant combined with another gene [34]. This *BRCA*-centered distribution, in contrast to ours, reflects the influence of the underlying population genetics and of the referral indication on the combinations detected. In that cohort, double heterozygotes did not show a more severe phenotype than single heterozygotes, including no difference in age at diagnosis, which contrasts with our findings; both results, however, rest on very small numbers of carriers.

A Canadian single-center cohort of 11,344 individuals undergoing hereditary cancer genetic testing identified 65 eligible double heterozygous carriers. The authors restricted the term MINAS to carriers of PVs in two autosomal-dominant CSGs, analyzing AD-AR combinations separately—an approach comparable to our D-D/D-R classification [35]. There, 28% of affected AD-AD carriers had at least one cancer not strongly associated with the underlying genes, a considerably higher proportion of atypical phenotypes than the single case (case 12) observed in our series. The authors also acknowledged that changing panel use over time and the absence of routine retesting precluded a reliable estimate of the true prevalence of MINAS, a limitation shared by our cohort.

The lack of association between the nature of inheritance of the variants and phenotypes was likely limited by the small number of cases and the absence of recessive–recessive cases, the combination with a stronger (not statistically significant) association with atypical phenotypes made in the review by Yuen at al. 2025 [5]. All calculations in this study should be interpreted as exploratory due to the small number of MINAS cases and the lack of correction for multiple comparisons.

Regarding clinical management, although no validated tools or recommendations are currently available to establish organ-specific cancer risk in patients with MINAS, these findings have implications for clinical practice [36]. Current evidence, derived primarily from case reports, small case series, and retrospective reviews, suggests that some individuals with MINAS may present with more severe phenotypes, including multiple primary malignancies and earlier cancer onset [5]. Accordingly, genetic counseling should address the uncertainty surrounding the combined cancer risk conferred by multiple pathogenic variants and consider the inheritance pattern of each gene involved, as this may influence recurrence risk assessment, reproductive counseling, and cascade testing of at-risk relatives [5]. In the absence of MINAS-specific management guidelines, surveillance in our unit was individualized in these individuals according to the cancer risks associated with each pathogenic variant, following the approach proposed by Yuen et al. [5], and cascade testing was offered to relatives at risk for both variants. In case 8, which involved a patient presenting with a more severe phenotype than expected for an *ATM* single-variant carrier, with early-onset breast and ovarian cancer, we proposed to the treating breast cancer team to consider risk-reducing mastectomy even in the absence of bilateral breast cancer in the family.

This study has several limitations that should be acknowledged. The observed gene distribution pattern may reflect a referral bias where not all patients with an indication for testing are yet being tested; analyses within the institution indicate that, during the same period between 2020 and 2026, testing was performed in only 10% of cases meeting NCCN criteria for testing in breast, ovarian, endometrial, pancreatic, colon, gastric, prostate, and renal cancers. Additionally, the number of genes analyzed evolved over the study period (Supplementary Table S2), with earlier panels including fewer genes than the current panel (ranging from approximately 40 to 113 genes nowadays), potentially underestimating the real prevalence of MINAS. Moreover, in this period 35.6% of studied cases had a result with a variant of uncertain significance (VUS), correlating with reports in Hispanic populations, where between 5 and 10% of cases are expected to be reclassified as a PV/LPV in the future [24]. An additional limitation of this study is the lack of a national population-based reference database of genetic variants in Chile, which precluded evaluation of the population frequency of the identified variants in an unselected Chilean population. Also, the relatively small sample size precludes definitive genotype–phenotype correlations. The number of cases identified has not allowed us, to date, to draw conclusions from the longitudinal follow-up of these individuals, nor to establish whether the surveillance strategy adopted detects malignancies beyond the spectrum expected for each variant considered separately, or whether it should be modified for particular gene combinations.

Despite these limitations our findings contribute to the limited body of literature on MINAS in Latin America. Future multicenter collaborative studies with larger cohorts will be essential to better characterize the clinical significance and management implications of MINAS in this population.

## Figures and Tables

**Table 1:** MINAS cases—genotypic and clinical description.

Case N	Gene 1	Gene 2	Sex	Phenotype	Previous Reports *
1	*BRCA1*	*CDKN2A*	F	TNBC at 51 yrs	Yes (same cohort)
2	*BRCA1*	*PMS2*	F	TNBC at 33 yrs	No
3	*BRCA2*	*FANCA*	F	HR+ breast cancer at 22 yrs	Yes
4	*BRCA2*	*ATM*	F	TNBC at 41 yrs	Yes
5	*BRCA2*	*MUTYH*	F	HR+ breast cancer at 32 yrs	Yes
6	*ATM*	*MLH1*	F	In situ HR breast cancer at 38 yrs and cecal MMRd stage IV cancer at 38 yrs	No
7	*ATM*	*MUTYH*	F	HR+ breast cancer at 46 yrs	Yes
8	*ATM*	*FANCM*	F	Breast cancer at 37 yrs and ovarian cancer at 41 yrs	No
9	*ATM*	*CHEK2*	F	HER2+ breast cancer at 29 yrs	Yes (same cohort)
10	*CDKN2A*	*FLCN*	F	Five primary melanomas, the first at age 40 and multiple nevi	No
11	*CDKN2A*	*LZTR1*	F	Melanoma at 34 yrs and multiple nevi	No
12	*CDKN2A*	*MUTYH*	F	TNBC at 44 yrs	No
13	*SDHB*	*RECQL4*	F	Paraganglioma at 17 yrs	No
14	*APC*	*MUTYH*	F	Family history of pancreatic cancer	Yes

TNBC: triple negative breast cancer; HR: hormone receptors; MMRd: mismatch repair deficient. * Previous reports described in Section 3.3.

**Table 2:** Genetic combinations in identified MINAS cases.

Case N	Gene 1	Variant HGVS	Gene 2	Variant HGVS	Gene 1/Gene 2 Mode of Inheritance
1	*BRCA1*	c.3759dup (p.Lys1254*)	*CDKN2A*	c.-34G>T	D-D
2	*BRCA1*	c.3331_3334del (p.Gln1111Asn fs*5)	*PMS2*	c.137G>T (p.Ser46Ile)	D-D
3	*BRCA2*	c.4740_4741dup (p.Glu1581Val fs*37)	*FANCA*	c.2639G>A (p.Arg880Gln)	D-R
4	*BRCA2*	c.6024dup (p.Gln2009Alafs*9)	*ATM*	c.8122G>A (p.Asp2708Asn)	D-D
5	*BRCA2*	c.9097dup (p.Thr3033AsnfsTer11)	*MUTYH*	c.1103G>A p.Gly368Asp	D-R
6	*ATM*	c.8122G>A (p.Asp2708Asn)	*MLH1*	c.588+5G>T	D-D
7	*ATM*	c.3381_3384del (p.Gln1128Lys fs*3)	*MUTYH*	c.1103G>A (p.Gly368Asp)	D-R
8	*ATM*	c.8319_8323dup (p.Pro2775Leu fs*33)	*FANCM*	c.5791C>T (p.Arg1931*)	D-R
9	*ATM*	c.2466+1G>A	*CHEK2*	c.445-2A>G	D-D
10	*CDKN2A*	c.-34G>T	*FLCN*	c.1658G>A (p.Trp553*)	D-D
11	*CDKN2A*	c.-34G>T	*LZTR1*	c.2387T>C (p.Ile796Thr)	D-D
12	*CDKN2A*	c.-34G>T	*MUTYH*	c.1103G>A (p.Gly368Asp)	D-R
13	*SDHB*	c.166_170del (p.Pro56Tyr fs*5)	*RECQL4*	c.2547_2548del (p.Phe850Pro fs*33)	D-R
14	*APC*	c.3920T>A (p.Ile1307Lys) *	*MUTYH*	c.452A>G (p.Tyr151Cys).	D-R

* The p.Ile1307Lys variant is considered a low-penetrance variant, common in the Ashkenazi Jewish population [12], and was reported by the laboratory as a likely pathogenic variant in this case.

**Table 3:** Previously reported cases sharing the MINAS gene combinations identified in this cohort.

Case	Gene Combination	Previous Report	Reported Phenotype, Age at Diagnosis	Phenotype in This Cohort
1	*BRCA1*/*CDKN2A*	Aguilar et al. 2025 [6]	TNBC, 30 yrs	TNBC, 51 yrs
		Ubilla et al. 2024 [13] †	TNBC, 51 yrs	
3	*BRCA2*/*FANCA*	LOVD MINAS_31 (Singapore)	TNBC, 44 yrs	HR+ breast cancer, 22 yrs
4	*BRCA2*/*ATM*	Tedaldi et al. 2017 [14]	Breast carcinoma in situ, 38 yrs; second intraductal breast carcinoma, 45 yrs	TNBC, 41 yrs
		Nurmi et al. 2019 [15]	Breast cancer, 76 yrs	
		Duzkale Teker and Eyerci (2021) [16]	Breast cancer, 34 yrs; pancreatic cancer, 48 yrs	
		van der Merwe et al. 2024 [17]	Invasive breast carcinoma, 39 yrs	
		Pócza et al. 2025 [18]	HR+ breast cancer, 30 yrs	
		Akcan et al. 2026 [19]	Two patients: bilateral breast cancer, 70 yrs; unilateral breast cancer, 68 yrs	
		Herold et al. 2026 [20]	Nine patients with breast cancer (GC-HBOC registry)	
		LOVD MINAS_08 (Brazil)	Breast cancer, 31 yrs	
		LOVD MINAS_18 (Singapore)	ER/PR+, HER2− IDC, 45 yrs	
5	*BRCA2*/*MUTYH*	Guindalini et al. 2022 [21]	TNBC, 33 yrs (Brazil)	HR+ breast cancer, 32 yrs
		Akcan et al. 2026 [19]	Two patients with breast cancer, 35 and 38 yrs	
		LOVD MINAS_24 (Singapore)	ER/PR+, HER2− IDC	
7	*ATM*/*MUTYH*	Agaoglu and Doganay 2021 [22]	Breast cancer, 30 yrs (Türkiye)	HR+ breast cancer, 46 yrs
9	*ATM*/*CHEK2*	Ubilla et al. 2024 [13] †	HER2+ breast cancer, 29 yrs	HER2+ breast cancer, 29 yrs
		Akcan et al. 2026 [19]	Two patients: breast cancer, 58 yrs; unaffected, 45 yrs	
		Herold et al. 2026 [20]	Twenty patients with breast cancer (GC-HBOC registry)	
14	*APC*/*MUTYH*	Akcan et al. 2026 [19]	Rectal cancer; unaffected patient, 21 yrs	No personal history of cancer; family history of pancreatic cancer

TNBC, triple-negative breast cancer; HR, hormone receptor; IDC, invasive ductal carcinoma; ER, estrogen receptor; PR, progesterone receptor; HER2, human epidermal growth factor receptor 2; LOVD, Leiden Open Variation Database; GC-HBOC, German Consortium for Hereditary Breast and Ovarian Cancer. † Corresponds to the same patient reported here, previously published as part of the same FALP cohort.

## Data Availability

The original contributions presented in this study are included in the article. Further inquiries can be directed to the corresponding author.

## Supplementary Materials

The following supporting information can be downloaded at: https://www.mdpi.com/article/10.3390/genes17070839/s1, Table S1: Hereditary cancer multi-gene panels used; Table S2: Gene content of hereditary cancer panels used during the study period; Supplementary Table S3: Demographic and clinical characteristics of the tested individuals; Supplementary Table S4: Age distribution of the tested individuals; Supplementary Table S5: Distribution of cancer type in the tested individuals with cancer.

## Abbreviations

The following abbreviations are used in this manuscript:

AD-AD	Autosomal Dominant–Autosomal Dominant
AD-AR	Autosomal Dominant–Autosomal Recessive
BC	Breast Cancer
CAP	College of American Pathologists
CI	Confidence Interval
CLIA	Clinical Laboratory Improvement Amendments
CSG	Cancer Susceptibility Gene/s
D-D	Dominant–Dominant
D-R	Dominant–Recessive
ER	Estrogen Receptor
FALP	Fundación Arturo López Pérez
FONASA	Fondo Nacional de Salud (Chilean national public health insurance)
GC-HBOC	German Consortium for Hereditary Breast and Ovarian Cancer
HBOC	Hereditary Breast and Ovarian Cancer
HER2	Human Epidermal Growth Factor Receptor 2
HGVS	Human Genome Variation Society
HR	Hormone Receptor
IDC	Invasive Ductal Carcinoma
IQR	Interquartile Range
LOVD	Leiden Open Variation Database
MINAS	Multilocus Inherited Neoplasia Alleles Syndrome
MMRd	Mismatch Repair Deficient
NCCN	National Comprehensive Cancer Network
NGS	Next-Generation Sequencing
OR	Odds Ratio
PR	Progesterone Receptor
PV/LPV	Pathogenic Variant/Likely Pathogenic Variant
R-R	Recessive–Recessive
TNBC	Triple Negative Breast Cancer
VUS	Variant of Uncertain Significance
WES	Whole-Exome Sequencing
WGS	Whole-Genome Sequencing
