# Supplementary material for: Report of Multilocus Inherited Neoplasia Alleles Syndrome in a Chilean Oncology Institute: New Combinations and Genetic Landscape
Source: Genes (Basel). 2026 Jul 22;17(7):839. doi: 10.3390/genes17070839 (PMC13410221; doi:10.3390/genes17070839)
Supplement: Supplementary file 1 [file genes-17-00839-s001.zip › genes-4382643-supplementary.pdf]

Supplementary tables

Supplementary Table S1. Hereditary cancer multi-gene panels used

Panel	n tests	Years used	Gene count	% of tests
Hereditary breast and gyn cancers (Invitae)	176	2020-2022	21	9.0%
Hereditary colorectal cancer (Invitae)	10	2021-2023	22	0.5%
Hereditary common cancers (Invitae)	28	2020-2023	49	1.4%
Breast/ovary (Mendelics)	145	2022-2024	38	7.4%
Multicancer customized (Mendelics)	891	2022-2024	84	45%
Multicancer gold standard (Mendelics)	552	2025-2026	100	28.1%
Hereditary oncomap (FALP)	130	2023-2026	113	6.6%
Other panels	30	2020-2026	Variable	1.5%

Supplementary Table S2. Gene content of hereditary cancer panels used during the study period

Panel	Gene content
Hereditary breast and gyn cancers (Invitae)	*ATM; BARD1; BRCA1; BRCA2; BRIP1; CDH1; CHEK2; DICER1; EPCAM; MLH1; MSH2; MSH6; NF1; PALB2; PMS2; PTEN; RAD51C; RAD51D; SMARCA4; STK11; TP53*
Hereditary colorectal cancer (Invitae)	*APC; AXIN2; BLM; BMPR1A; EPCAM; GREM1; MBD4; MLH1; MSH2; MSH3; MSH6; MUTYH; NTHL1; PMS2; POLD1; POLE; PTEN; RPS20; SMAD4; STK11; TP53*
Hereditary common cancers (Invitae)	*APC; ATM; AXIN2; BAP1; BARD1; BMPR1A; BRCA1; BRCA2; BRIP1; CDH1; CDK4; CDKN2A; CHEK2; CTNNA1; DICER1; EPCAM; FH; GREM1; HOXB13; KIT; MBD4; MEN1; MLH1; MSH2; MSH3; MSH6; MUTYH; NF1; NTHL1; PALB2; PDGFRA; PMS2; POLD1; POLE; PTEN; RAD51C; RAD51D; RPS20; SDHA; SDHB; SDHC; SDHD; SMAD4; SMARCA4; STK11; TP53; TSC1; TSC2; VHL*
Breast/ovary (Mendelics)	*APC; ATM; BAP1; BARD1; BLM; BMPR1A; BRCA1; BRCA2; BRIP1; CDH1; CDK4; CDKN2A; CHEK2; EGFR; EPCAM; FANCC; FANCM; MEN1; MET; MLH1; MSH2; MSH3; MSH6; MUTYH; NBN; NTHL1; PALB2; PMS2; POLD1; POLE; PTEN; RAD51C; RAD51D; RECQL; RET; STK11; TP53*
Multicancer customized (Mendelics)	*AIP; ALK; APC; ATM; AXIN2; BAP1; BARD1; BLM; BMPR1A; BRCA1; BRCA2; BRIP1; CASR; CDC73; CDH1; CDK4; CDKN1B; CDKN1C; CDKN2A; CEBPA; CHEK2; CTNNA1; DICER1;*

Panel	Gene content
	DIS3L2; EGFR; EPCAM; FH; FLCN; GATA2; GPC3; GREM1; HOXB13; HRAS; KIT; MAX; MEN1; MET; MITF; MLH1; MSH2; MSH3; MSH6; MUTYH; NBN; NF1; NF2; NTHL1; PALB2; PDGFRA; PHOX2B; PMS2; POLD1; POLE; POT1; PRKAR1A; PTCH1; PTEN; RAD50; RAD51C; RAD51D; RB1; RECQL4; RET; RUNX1; SDHA; SDHAF2; SDHB; SDHC; SDHD; SMAD4; SMARCA4; SMARCB1; SMARCE1; STK11; SUFU; TERC; TERT; TMEM127; TP53; TSC1; TSC2; VHL; WRN; WT1.
Multicancer gold standard (Mendelics)	*AIP; AKT1; ALK; APC; ATM; ATP4A; ATR; AXIN2; BAP1; BARD1; BLM; BMPR1A; BRCA1; BRCA2; BRIP1; CDC73; CDH1; CDK12; CDK4; CDKN1B; CDKN1C; CDKN2A; CEBPA; CHEK1; CHEK2; CTNNA1; DICER1; DIS3L2; EGFR; EPCAM; FANCA; FANCC; FANCL; FANCM; FH; FLCN; GATA2; GPC3; GREM1; HOXB13; IPMK; KIT; LZTR1; MAX; MBD4; MEN1; MET; MITF; MLH1; MRE11; MSH2; MSH3; MSH6; MUTYH; NBN; NF1; NF2; NTHL1; PALB2; PDGFRA; PHOX2B; PIK3CA; PMS2; POLD1; POLE; POT1; PRKAR1A; PTCH1; PTEN; RABL3; RAD50; RAD51; RAD51B; RAD51C; RAD51D; RB1; RECQL; RECQL4; RET; RNF43; RPS20; RUNX1; SDHA; SDHAF2; SDHB; SDHC; SDHD; SMAD4; SMARCA4; SMARCB1; SMARCE1; STK11; SUFU; TERT; TMEM127; TP53; TSC1; TSC2; VHL; XRCC2*
Hereditary oncomap	*ABRAXAS1; ACD; AIP; AKT1; APC; ATM; BAP1; BARD1; BLM; BMPR1A; BRCA1; BRCA2; BRIP1; CASR; CDC73; CDH1; CDK4; CDKN1B; CDKN2A; CEBPA; CHEK2; CTRC; DDB2; DICER1; DIS3L2; EPCAM; ERCC1; ERCC2; ERCC3; ERCC4; ERCC5; FANCA; FANCB; FANCC; FANCD2; FANCE; FANCF; FANCG; FANCI; FANCL; FANCM; FH; FLCN; GALNT12; GATA2; GPC3; GREM1; HOXB13; KIF1B; KIT; LZTR1; MAX; MEN1; MET; MITF; MLH1; MRE11A; MSH2; MSH3; MSH6; MUTYH; NBN; NF1; NF2; NSD1; NTHL1; PALB2; PDGFRA; PHOX2B; PIK3CA; PMS2; POLD1; POLE; POT1; PRKAR1A; PTCH1; PTEN; RAD50; RAD51; RAD51B; RAD51C; RAD51D; RB1; RECQL4; RET; RHBDF2; RINT1; RUNX1; SDHA; SDHAF2; SDHB; SDHC; SDHD; SLX4; SMAD4; SMARCA4; SMARCB1; SMARCE1; SPINK1; SPRED1; STK11; SUFU; TERF2IP; TERT; TMEM127; TP53; TSC1; TSC2; VHL; WT1; XPA; XPC; XRCC2*

Supplementary Table S3. Demographic and clinical characteristics of the tested individuals

Characteristic	n	%
Total individuals tested	1962	100%
Individuals with cancer	1634	83.3%
Female	1864	95.0%
FONASA coverage	1236	63.0%

Supplementary Table S4. Age distribution of the tested individuals

Age group at assessment (years)	n	%
10-19	7	0.4%
20-29	67	3.4%
30-39	484	24.7%
40-49	739	37.7%
50-59	378	19.3%
60-69	199	10.1%
70-79	78	4.0%
80-89	10	0.5%

Supplementary Table S5. Distribution of cancer type in the tested individuals with cancer

Diagnosis / tumor category	n	% of tested cohort
Breast	1318	67.2%
Prostate	66	3.4%
Colon	62	3.2%
Ovary	59	3.0%
Kidney	24	1.2%
Pancreas	23	1.2%
Endometrial	19	1.0%
Thyroid	18	0.9%
Gastric	16	0.8%
Melanoma	14	0.7%
Skin non-melanoma	8	0.4%
Lung	8	0.4%
Cervical	4	0.2%
Paraganglioma	4	0.2%
Hematology	4	0.2%
Sarcoma	4	0.2%
Liver	2	0.1%
Brain	1	0.1%
Neuroendocrine	1	0.1%
Retroperitoneal	1	0.1%
Biliary tract	1	0.1%
Urinary tract	1	0.1%
Other	72	3.7%
Benign tumour	54	2.8%
